# Determining the Sulfate Content in Phosphogypsum and Cement-Based Materials Based on Conductivity Titration

**DOI:** 10.3390/ma18163758

**Published:** 2025-08-11

**Authors:** Dafu Wang, Jieming Zhang, Jingting Zhou, Yudong Sun, Jun Ren, Xincheng Li, Zhiyong Liu

**Affiliations:** 1 School of Architecture and Planning, Yunnan University, Kunming 650050, China; 20230107@ynu.edu.cn (D.W.); zhangjieming_bl14@stu.ynu.edu.cn (J.Z.); zhoujingting@stu.ynu.edu.cn (J.Z.); yudong.sun@outlook.com (Y.S.); renjunking@aliyun.com (J.R.); 2 Yunnan Academy of Building Research, Kunming 650223, China; 3 School of Materials Science and Engineering, Southeast University, Nanjing 211189, China; liuzhiyong0728@163.com

**Keywords:** conductivity titration, sulfate content, phosphogypsum, cement-based materials, internet of things

## Abstract

Accurate determination of sulfate content in phosphogypsum (PG) and cement-based materials is crucial for understanding the corrosion mechanisms of cement-based materials, developing corrosion models, establishing durability design methods, and implementing maintenance strategies. To overcome the limitations of traditional gravimetric and EDTA titration methods in accurately quantifying low-concentration SO_4_^2−^ in PG and cement-based materials, an IoT-enabled conductometric titration system was developed to improve precision and automation. First, the principle of conductivity titration is introduced, in which Ba(NO_3_)_2_ is used as the titrant. Second, a method for eliminating the effects of H^+^, Cl^−^, and Ca^2+^ ions is proposed. The impact of the titration rate, volume of liquid to be measured, titrant concentration, and other interfering ions on the results is discussed. Finally, the conductivity titration method was successfully applied to determine sulfate content in PG and cement-based materials. The results demonstrate that the self-developed conductivity titrator exhibits high testing accuracy, with a standard deviation of 0.013 for 15 repeated titrations, a coefficient of variation of 0.52%, and a recovery rate between 103.2% and 103.9%. The optimal solution volume to be determined was 5 mL. Ba(NO_3_)_2_, at approximately twice the sulfate concentration, enhances endpoint sensitivity and minimizes precipitation interference. Ag_2_O and CO_2_ significantly reduce the interference from H^+^, Cl^−^, and Ca^2+^ ions by generating weakly conductive substances, such as H_2_O, AgCl, Ag_3_PO_4_, CaF_2_, and CaCO_3_. Conductometric titration demonstrated accurate SO_4_^2−^ quantification in PG and cement-based materials, enabling standardized protocols. This approach provides both theoretical and technical support for rapid sulfate detection in complex systems, with significant implications for both industry and academia. For the industry, it offers a reliable and standardized method for sulfate detection, enhancing quality control and process efficiency. For academia, it establishes a foundation for further research in civil engineering and environmental material analysis, contributing to both practical applications and theoretical advancements.

## 1. Introduction

Phosphogypsum (PG) is a prevalent industrial by-product generated during the wet-process production of phosphoric acid. Its main component is calcium sulfate dihydrate (CaSO_4_⋅2H_2_O), accompanied by trace amounts of undecomposed phosphate rock, free phosphoric acid, fluoride, and heavy metal impurities [1]. As global demand for phosphate fertilizers increases, the continuous accumulation of PG stockpiles occupies land resources. It poses a potential threat to the ecological environment due to its soluble sulfate and acidic substances. With the rising global demand for phosphate fertilizers, the accumulation of PG stockpiles is becoming increasingly problematic, occupying land resources and posing environmental risks due to its soluble sulfates and acidic constituents.

In recent years, the utilization of PG as a secondary resource has garnered growing attention, particularly in the production of cement retarders, gypsum-based building products [2,3], and road-based materials [4]. Accurate determination of sulfate ions in PG and PG-based cementitious systems is essential, as sulfate content directly influences hydration kinetics, mechanical properties, and long-term durability [5]. Sulfate attacks remain a significant cause of durability degradation in concrete structures [6]. They involve complex chemical interactions between external sulfate ions and the hydration products of cement [7,8]. Therefore, a rapid and reliable method for quantifying sulfate content is crucial, not only for assessing material durability but also for developing predictive corrosion models and improving structural design against sulfate exposure [9].

In the construction and civil engineering industries, traditional methods for determining sulphate ions, such as the gravimetric method [10,11] and EDTA titration [12], are widely used in cement-based material testing. However, these techniques have significant limitations. The gravimetric method yields large errors at low sulphate concentrations. It is operationally complex, while the EDTA titration method, which uses chromothion blue T as an indicator, is prone to interference from Ca^2+^ and Mg^2+^ ions in the solution, making the endpoint color change difficult to identify, resulting in significant errors [13,14]. In summary, due to the distinct characteristics of SO_4_^2−^ content testing in PG and cement-based materials, traditional methods are difficult to adapt, and it is imperative to seek new testing methods with high accuracy, good adaptability, and fast speed.

As an ionic testing method, conductivity titration is applied in various fields due to its high efficiency and convenience [15,16,17,18], but its use in civil engineering remains limited. The study aims to address this gap by investigating the application of conductometric titration for the rapid and accurate measurement of SO_4_^2−^ in PG and cement-based materials. This study introduces the principle of conductometric titration for the analysis of PG and cement-based materials. It presents a detailed description of a custom-designed conductometric titration system developed for this purpose. The stability and analytical accuracy of the system are systematically evaluated. In addition, effective strategies are proposed to eliminate interference from H^+^, Cl^−^, and Ca^2+^ ions during the titration process. Optimal titration parameters are established, and the method is successfully applied to determine sulfate content in PG and cement-based materials. The findings are expected to provide valuable technical support for quality control and performance optimization in sulfate-bearing cementitious materials, as well as reliable data for durability assessment and design in sulfate-rich environments. Furthermore, this work may serve as a valuable reference for the broader application of conductometric titration techniques in complex material systems.

## 2. Conductivity Titration Theory

The principle of conductivity titration, a fundamental concept in analytical chemistry, is based on the fact that each ion in an electrolyte solution contributes to its overall conductivity [19,20,21]. When electrolyte solutions are mixed, reactions such as precipitation, redox, or neutralization may occur, altering ion concentrations and thus affecting the conductivity of the system. In conductivity titration, the gradual addition of a titrant changes the ionic composition of the solution. The endpoint of titration is the point at which the slope of the change in conductivity with titration volume undergoes a sudden change [22].

**Figure 1:**
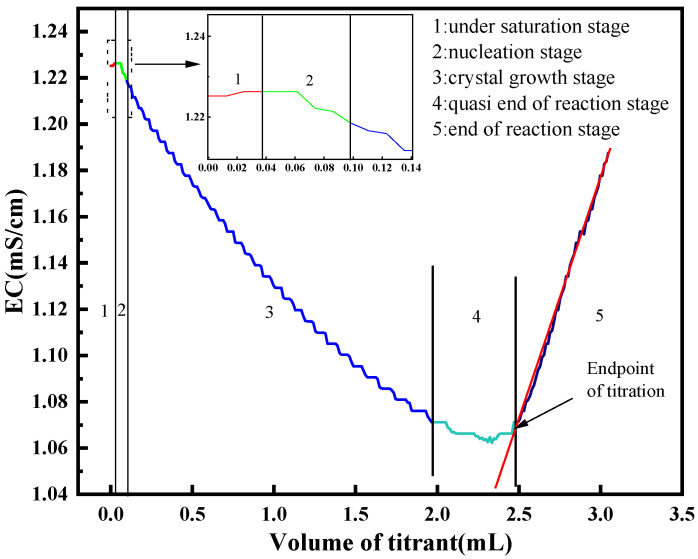
Typical curve of solution conductivity with the volume of titrant.

Conductometric titration is an electrochemical technique in which the endpoint is determined by monitoring the abrupt change in the conductivity of the reaction mixture. For precipitation reactions, such as the titration of SO_4_^2−^ with Ba^2+^, the conductivity curve typically follows six distinct stages, as illustrated in Figure 1: (1) Undersaturation stage: The added Ba^2+^ and existing SO_4_^2−^ remain below the solubility product; both exist as free ions, leading to an increase in conductivity. (2) Crystallization nucleation stage: The ion product exceeds the solubility limit, initiating BaSO_4_ precipitation. The rate of conductivity increase slows due to the removal of free ions. (3) Crystal growth stage: Ba^2+^ and SO_4_^2−^ react completely, resulting in a net decrease in conductivity as ionic species are consumed. (4) Quasi-end of reaction stage: Residual SO_4_^2−^ reacts with additional Ba^2+^, further decreasing conductivity, but at a slower rate. (5) End of reaction stage: Excess Ba^2+^ remains unreacted in ionic form, causing the conductivity to increase again. (6) Infinite titration stage: Continued addition of Ba(NO_3_)_2_ causes the conductivity to approach that of the pure titrant gradually.

Conductivity titration offers a precise and reliable method for determining the ion concentration in a solution. This process involves monitoring the dynamic change in conductivity of a mixed solution during the titration. The sudden change in conductivity is then identified to determine the endpoint of the titration. This information accurately determines the concentration of specific ions in the solution.

In typical SO_4_^2−^ titrations, BaCl_2_ is often used as the titrant. However, in the presence of Ag^+^, Cl^−^ can form AgCl precipitates, interfering with endpoint detection. To avoid such complications, Ba(NO_3_)_2_ was used in this study. Its reaction with SO_4_^2−^ forms BaSO_4_, producing a clear and distinct conductivity change that facilitates accurate endpoint identification.

## 3. Materials and Methods

### 3.1. Conductivity Titrators

**Figure 2:**
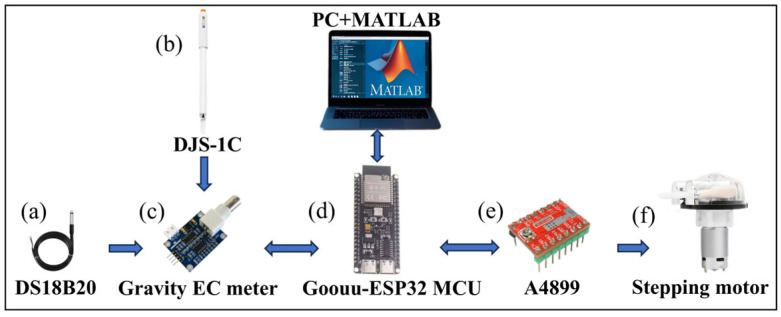
Composition of the self-designed conductive titrator [25]. (**a**) DS18B20; (**b**) DJS−1C; (**c**) Gravity EC meter; (**d**) Goouuu−ESP32; (**e**) A4899; (**f**) Stepping motor.

In the early stages of electrochemical titration research, numerous conductivity titrators and associated methodologies were developed by investigators [23,24]. However, these systems exhibit limited accuracy and reproducibility, primarily attributed to the insufficient sensitivity of electrochemical sensors, the low resolution of signal acquisition systems, and inherent methodological deficiencies in endpoint determination protocols. Such technical constraints substantially hinder the reliability of experimental outcomes in precise quantitative analyses. For this reason, new conductivity titrators and titration systems have been designed and developed. The titration system is shown in Figure 2, which mainly consists of a goouuu-ESP32 MCU, conductivity electrode DJS, EC drive module, 42 stepper motor, DS18B20, A4899 stepper motor drive module, magnetic stirrer, and 6-roller peristaltic pump.

#### 3.1.1. Goouuu-ESP32 MCU

The Goouuu-ESP32 MCU (Ismart Electronic Co., Ltd., Shenzhen, China) is a powerful microcontroller module based on the ESP32 chip from Lexin for various IoT and embedded applications, as shown in Figure 2d. In the conductivity titrator, analog pins are used to precisely control the output of the conductivity driver module and collect the voltage signal fed back from the module in real time to calculate the conductivity of the solution. The digital pin is used to connect the temperature sensor and obtain the solution temperature data. Through the linkage of the A4899 stepper motor driver module (Yong Fukang Technology Co., Ltd., Shenzhen, China), the 42 stepper motors are precisely driven to complete the control of the liquid volume, thereby realizing the automation of the titration process.

#### 3.1.2. Conductivity Sensor

The conductivity sensor used is the DJS series conductivity electrode produced by the Leici firm, with a driver of the Gravity EC meter, as shown in Figure 2b,c. The measurement range of the commonly used conductivity electrode of DJS-0.1C is about 0–0.2 mS/cm, the measurement range of DJS-1C is about 0–20 mS/cm, and the measurement range of DJS-10C is about 2–200 mS/cm. To ensure accurate conductivity measurements, electrodes with appropriate cell constants should be selected based on the concentration range of the solution.

#### 3.1.3. A 42-Step Motor

A 42-step motor with six rollers driven by the A4899 module performs automatic titration during conductivity titrations, as shown in Figure 2e,f. The rotating rollers cyclically compress a flexible tube filled with liquid to control the volume of the titration solution and its flow rate.

#### 3.1.4. Temperature Sensor

Since the titration process is sensitive to temperature, and fluctuations in temperature can significantly affect conductivity measurements, a DS18B20 temperature sensor (Shenzhen RBD Sensor Technology Co., Ltd., Shenzhen, China) was integrated into the system, as shown in Figure 2a, to monitor the solution temperature in real time. If the solution reaches the target temperature, the titration proceeds. Otherwise, a temperature correction is applied using a programmed calibration equation on the computer to ensure consistency in test conditions. The correction equation is presented in Equation (1).

(1) $$\Lambda =\Lambda_{25^{\circ }C}\left(1.0+0.0185\left(T_{test}-25.0\right)\right)$$

#### 3.1.5. CO_2_ Generator

**Figure 3:**
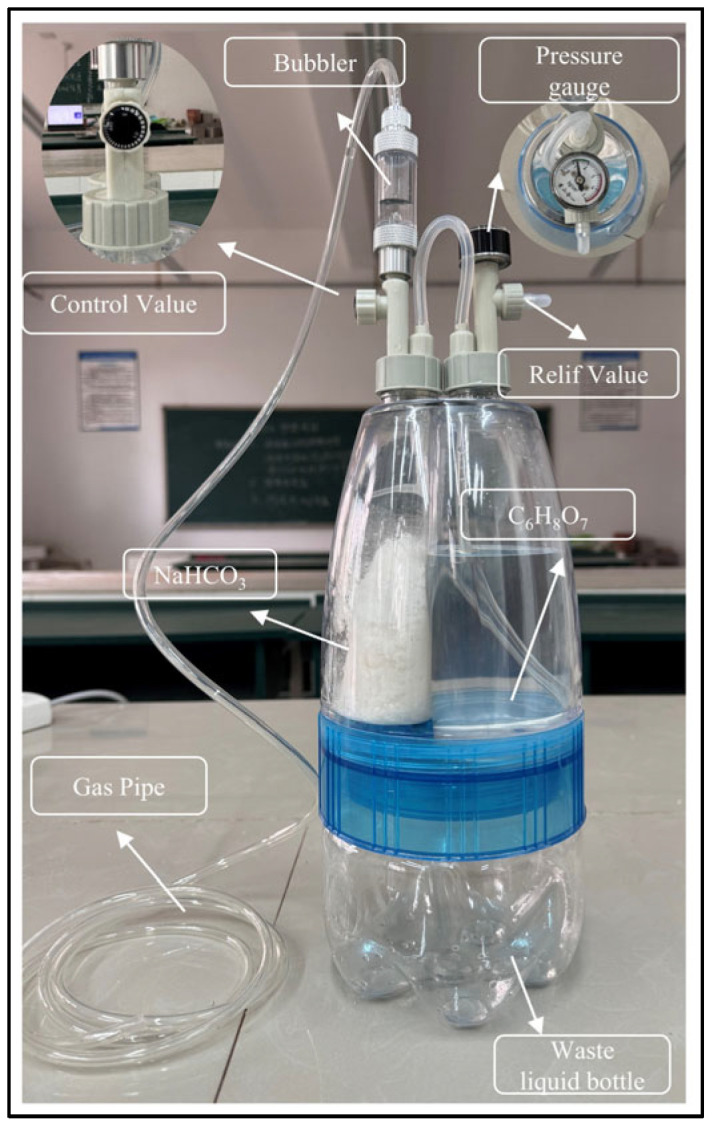
Carbon dioxide generator.

A CO_2_ generator is employed to eliminate interfering ions such as H^+^, Cl^−^, and Ca^2+^ prior to conductivity titration. The configuration of the low-pressure CO_2_ generator is illustrated in Figure 3. It primarily comprises a pressure relief valve, pressure gauge, bubbler, control valve, outlet tube, sodium bicarbonate bottle, acid bottle, and waste container. The generator operates based on a pressure balance mechanism: the pressure differential between the acid reservoir and the sodium bicarbonate reaction chamber drives a self-regulating cycle of acid inflow and CO_2_ generation. The reaction between C_6_H_8_O_7_ and NaHCO_3_ that produces CO_2_ is shown in Equation (2).

(2) $$C_{6}H_{8}O_{7}+{NaHCO}_{3}⇋C_{6}H_{7}O_{7}{Na}_{3}+H_{2}O+{CO}_{2}↑$$

#### 3.1.6. Other Tools

**Figure 4:**
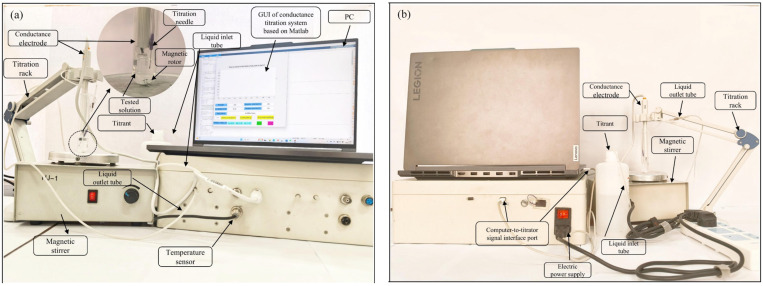
Composition of the conductive titration system. (**a**) front view; (**b**) rear view.

The composition of the conductive titration system is shown in Figure 4. Other tools include magnetic stirrers, an electronic balance with a range of 200 g and a sensitivity of 0.0001 g, volumetric flasks of 100 mL and 1000 mL, a triangular flask with a volume of 50 mL, brown glass bottles with volumes of 1000 mL and 100 mL, a transparent glass bottle with a volume of 1000 mL, measuring cylinders with ranges of 50 mL and 100 mL, a centrifuge tube with a volume of 20 mL, a plastic bottle with a volume of 50 mL, equipped with an asbestos mesh electric furnace, quantitative filter paper, dropper, pH test paper, beaker, etc.

### 3.2. Software for Conductivity Titration

**Figure 5:**
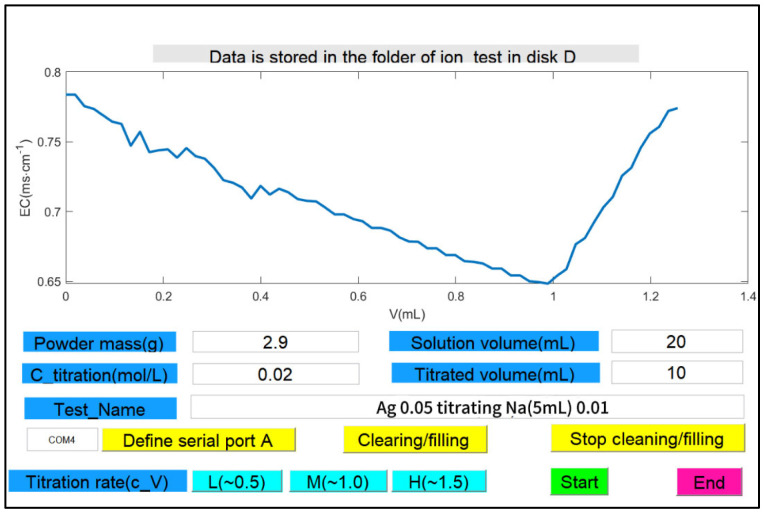
GUI interface of the titration system.

The titration system software comprises the operating system and control program. The operating system is based on the Arduino IDE2.3.2 (Arduino SA, Monza, Italy), which was developed in the C language. The Goouuu-ESP32 MCU is responsible for collecting conductivity and temperature data, controlling the stepper motor for titration, and transmitting data to the computer through the serial port. The control program was developed using MATLAB (R2022b) GUI and communicated with the ESP32 serial port through a dedicated support package to achieve the data acquisition and titration control shown in Figure 5. During operation, the device was connected, and the test could be started after inputting the powder mass, dissolved liquid volume, Ba(NO_3_)_2_ concentration, the liquid volume to be measured, the name of the sample, and the serial number of the computer, and the test data were automatically saved in the specified disk in the ‘Ion_test’ file.

### 3.3. Preparation of Solutions

A total of 13.07 g of Ba(NO_3_)_2_ was weighed and dissolved in deionized water to a final volume of 1 L, obtaining a 0.05 mol/L solution. A titrant of 0.02, 0.05, or 0.1 mol/L was selected according to the concentration of sulfate. A total of 110 mL of concentrated hydrochloric acid was diluted to 1 L, making a 1.3 mol/L HCl solution. A total of 0.1 g of phenolphthalein was dissolved in ethanol and concentrated to 100 mL to prepare a phenolphthalein solution. A total of 1.42 g of Na_2_SO_4_ was dissolved in deionized water and concentrated to 1 L, resulting in a 0.01 mol/L solution. All solutions were mixed thoroughly and stored correctly.

### 3.4. Conductivity Titration Procedure

#### 3.4.1. Conditions for Conductivity Titration

During sulfate ion titration, the room temperature was maintained at 20 °C to 25 °C, and the humidity was controlled at 50 ± 10%. The temperatures of the standard and test solutions were kept within the range of 20 °C to 25 °C [26].

#### 3.4.2. Conductivity Electrode Calibration

To ensure the electrodes were in proper working condition, the conductive electrodes were calibrated according to the following steps: (1) The titrator was started, and the inlet of the buret was immersed in water. The “clean/fill” button on the GUI was clicked, and the water was allowed to flow out of the needle. The inlet was then emptied of water, and the peristaltic pump was kept running until the water in the tube was drained out, repeating the procedure three times. (2) A total of 5 mL of standard conductivity solution was taken in a 10 mL measuring cylinder and placed in a centrifuge tube. The standard conductivity solution used was a 0.01 mol/L KCl solution, with a conductivity of approximately 12.88 mS/cm at 25 °C. (3) The ethanol-cleaned electrode was suspended vertically in the solution, ensuring it was completely submerged and that the temperature sensor was synchronized with the current water temperature. (4) The “Start” button on the GUI was clicked to begin the test. The average of 100 data points was taken, and the ratio of the average to the standard deviation was calculated as the correction factor. (5) The correction factor was updated in the titration program to the current measured value. (6) The electrode was replaced or recalibrated after 72 h of accumulated use.

#### 3.4.3. Titration Volume Correction

To account for errors caused by environmental changes, usage scenarios, and other factors, a volumetric calibration of the instrument was performed before each test to ensure it was in optimal working condition. The titration volume correction involved the following steps: (1) Step (1) from Section 3.4.2 was followed, and the burette was then filled with the titrant. (2) A total of 5 mL of 0.01 mol/L Na_2_SO_4_ standard solution was measured into the centrifugal tube. (3) A magnetic stirrer was placed into the centrifugal tube, and the tube was positioned at the center of the stirrer. (4) Step (3) from Section 3.4.2 was followed, and the burette was placed into the centrifuge tube below the page. (5) The magnetic stirrer was started to ensure that the magnetic rotor did not touch the collision process during stirring. (6) The “Start” button was clicked, and the conductivity titration curve was observed. When noticeable changes in the conductivity titration curve were observed, and the curve showed a linear change trend, titration continued with 0.1–0.2 mL of titrant. (7) The “Send” button was clicked to end the test according to Step (5) from Section 3.4.2.

The titration volume was obtained from the point where the extension of the linearly varying segment at the late stage of the titration was tangent to the titration curve. The result was retained to three decimal places. The ratio of the theoretically expected volume of 1 mL to the titration volume of Ba(NO_3_)_2_ measured by the test was used as the titration volume correction factor. The correction factor was updated in the titration procedure to the current measured value.

#### 3.4.4. Calculation of Sulfate Ion Test Results

The sulfate ion content was calculated according to the following Equation (3):

(3) $$W_{{{SO}_{4}}^{2-}}^{w}=C_{Ba{\left({NO}_{3}\right)}_{2}}\frac{V_{Ba{\left({NO}_{3}\right)}_{2}}}{V_{2}}V_{1}^{w}\times 10^{-3}\times 96\times \frac{1}{m}\times 100$$

where $W_{{{SO}_{4}}^{2-}}^{w}$ is the sulphate ion content (%), retained to 3 decimal places, $C_{Ba{\left({NO}_{3}\right)}_{2}}$ is the concentration of barium nitrate standard solution (mol/L), $V_{Ba{\left({NO}_{3}\right)}_{2}}$ is the amount of barium nitrate standard solution (mL), $V_{1}^{w}$ is the amount of acid used to dissolve powder (mL), $V_{2}$ is the volume of solution to be measured (mL), and m is the mass of dissolved powder (g).

## 4. Results and Discussion

### 4.1. Precision and Stability of Conductivity Titration

**Figure 6:**
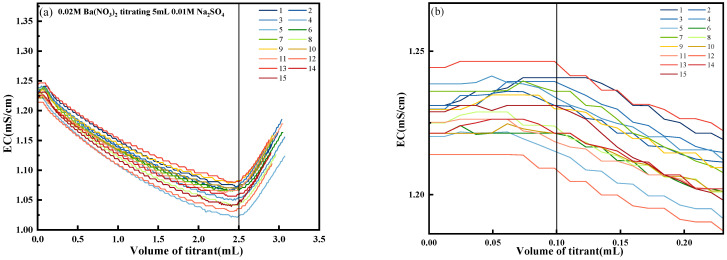
Change in solution conductivity with the drop volume of barium nitrate. (**a**) 15 series of titration curves; (**b**) Enlarged view of the starting point.

To further investigate the accuracy and stability of the conductivity titration method, it was validated via a recovery experiment and 15 sets of repeatability tests. In the repeatability tests, a 0.02 mol/L Ba(NO_3_)_2_ solution was used to titrate 5 mL of a 0.01 mol/L Na_2_SO_4_ solution, and the results of 15 repeated titrations are shown in Figure 6. Across 15 titration curves, the changes in this region exhibited a consistent magnitude.

As illustrated in Figure 6b, the initial conductivity of the solution ranged from 1.214 to 1.244 mS/cm. During the titration process, the dividing point between the undersaturation and nucleation stages on the titration curve was indistinct. However, when approximately 0.1 mL of titrant was added, the transition from the nucleation to the crystal growth stage became evident.

**Figure 7:**
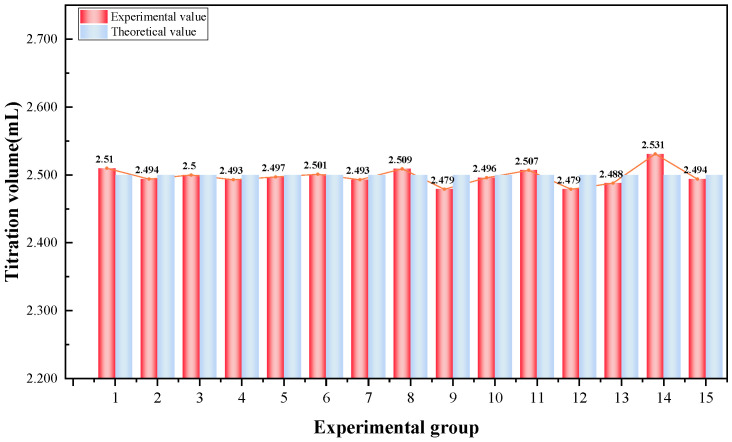
The results of 15 repeated titration experiments.

As shown in Figure 7, the comparison between the actual and theoretical values from 15 titration trials reveals a high degree of consistency, indicating strong data stability. The average titration volume of Ba(NO_3_)_2_ was 2.498 mL, with a standard deviation of 0.013 mL, a coefficient of variation of 0.52%, and an average relative error of 0.32%. Compared with the 1.13% relative error reported by Garcia’s conductometric titration method [27] and the significantly higher 6.4% error reported by Anechiţei [28], the self-developed conductivity titration system exhibits significantly higher titration accuracy.

**Figure 8:**
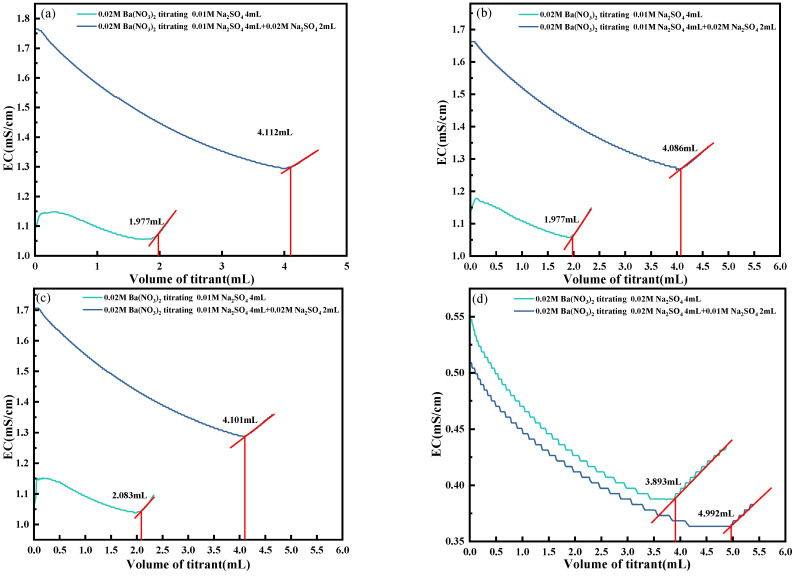

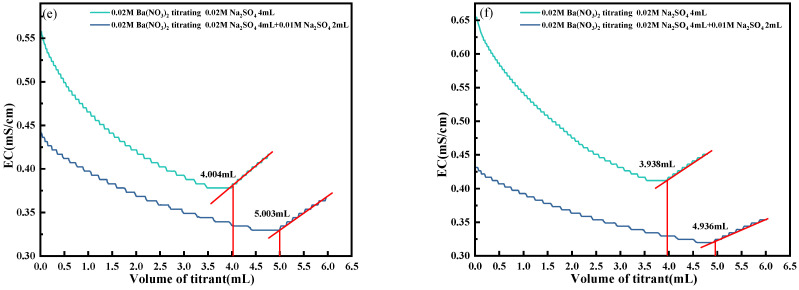
Recovery experiment. (**a**) The first experiment of Group A; (**b**) The second experiment of Group A; (**c**) The third experiment of Group A; (**d**) The first experiment of Group B; (**e**) The second experiment of Group B; (**f**) The third experiment of Group B.

A 0.02 mol/L Ba(NO_3_)_2_ solution was used as the titrant in the recovery experiment. Two experimental groups, A and B, were designed. Group A titrated unspiked samples consisting of 4 mL of 0.01 mol/L Na_2_SO_4_, while Group B titrated spiked samples consisting of 4 mL of 0.02 mol/L Na_2_SO_4_. For the spiking process, 2 mL of 0.02 mol/L Na_2_SO_4_ was added to Group A, and 2 mL of 0.01 mol/L Na_2_SO_4_ was added to Group B. The experimental results are shown in Figure 8. The recovery was calculated using Equation (4):

(4) $$A\left(\%\right)=\frac{(V_{2}-V_{1)}\times C_{1}}{C_{2}\times V_{3}}$$

where *C*_1_ is the concentration of titrant (mol/L), *C*_2_ is the concentration of the solution used for labeling (mol/L), *V*_1_ is the volume of the solution used for calibration (L), *V*_2_ is the volume of titrant consumed for the spiked solution (L), *V*_3_ is the volume of titrant consumed for the unspiked solution (L).

The recovery experiment results presented in Table 1 show a close agreement between the theoretical and measured values. In Group A, the theoretical titration volumes were 2 mL for the unspiked sample, which contained 4 mL of 0.01 mol/L Na_2_SO_4_, and 4 mL for the spiked sample, which was prepared by adding 2 mL of 0.02 mol/L Na_2_SO_4_ to the unspiked sample. The measured volume increment was 2.079 mL, corresponding to a recovery rate of 103.9%. In Group B, the theoretical volumes were 4 mL for the unspiked sample, composed of 4 mL of 0.02 mol/L Na_2_SO_4_, and 5 mL for the spiked sample, obtained by adding 2 mL of 0.01 mol/L Na_2_SO_4_. The measured increment was 1.032 mL, yielding a recovery of 103.2%. Both values fall within the acceptable range of 95–105% and are close to the ideal 100% recovery, confirming the high accuracy of the self-developed conductivity titration system in recovery testing.

### 4.2. Effect of the Solution Volume to Be Measured

Selecting an appropriate volume of the sample solution prior to titration is essential for achieving accurate titration results. To investigate this, a series of experiments was conducted using Ba(NO_3_)_2_ solutions at concentrations of 0.01 mol/L, 0.02 mol/L, 0.05 mol/L, and 0.1 mol/L to titrate 0.01 mol/L Na_2_SO_4_ solutions, with sample volumes set at 5 mL, 7.5 mL, and 10 mL.

**Figure 9:**
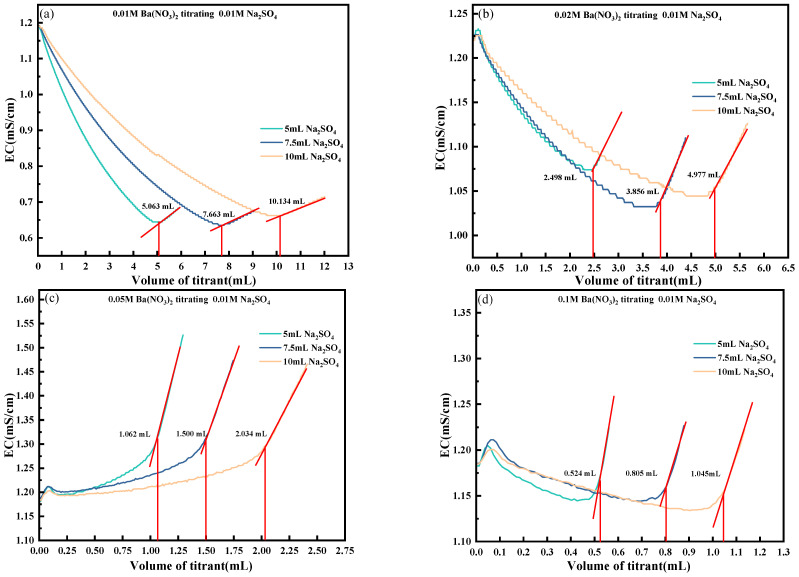
Change in conductivity with the volume of titrant under different titrant concentrations and different titration volumes. (**a**) 0.01 M Ba(NO_3_)_2_ titrating 0.01 M Na_2_SO_4_; (**b**) 0.02 M Ba(NO_3_)_2_ titrating 0.01 M Na_2_SO_4_; (**c**) 0.05 M Ba(NO_3_)_2_ titrating 0.01 M Na_2_SO_4_; (**d**) 0.1 M Ba(NO_3_)_2_ titrating 0.01 M Na_2_SO_4_.

As illustrated in Figure 9, when titrating solutions of varying volumes, smaller sample volumes exhibited a lower slope in the initial conductivity–titrant volume curve and a steeper slope near the endpoint, facilitating a more precise identification of the titration endpoint. For a given volume of titrant, higher concentrations of Ba(NO_3_)_2_ consumed more SO_4_^2−^ ions, accelerating the reaction. As the sample volume increased, the conductivity response transitioned from an initially linear behavior to a more complex, nonlinear pattern. When the sample volume exceeded a certain threshold, the effect of the titrant on the overall conductivity of the system became more pronounced, complicating endpoint determination.

As shown in Figure 9a, the titration of 5 mL, 7.5 mL, and 10 mL of 0.01 mol/L Na_2_SO_4_ solution using 0.01 mol/L Ba(NO_3_)_2_ exhibited a consistent and interpretable conductivity response. Upon the gradual addition of Ba(NO_3_)_2_, Ba^2+^ ions reacted with SO_4_^2−^ to form insoluble BaSO_4_, resulting in a reduction in the ionic concentration and, consequently, a decline in the solution’s conductivity. The titration endpoints for the 5 mL, 7.5 mL, and 10 mL Na_2_SO_4_ solutions were identified at 5.063 mL, 7.663 mL, and 9.993 mL, respectively. Beyond the endpoint, further addition of Ba^2+^ increased the ionic concentration, causing a subsequent rise in conductivity. Figure 9b presents the results obtained using 0.02 mol/L Ba(NO_3_)_2_ as the titrant, where the corresponding endpoints were 2.498 mL, 3.856 mL, and 5.003 mL for the same Na_2_SO_4_ solution volumes. The higher titrant concentration led to a faster reaction rate and a steeper change in conductivity near the equivalence point, making the inflection point more distinguishable. Similar trends were observed with titrant concentrations of 0.05 mol/L and 0.1 mol/L, though the titrant volumes required to reach the endpoints were significantly reduced due to the higher molar concentration of Ba^2+^. The accelerated reaction rate further enhanced the conductivity gradient around the endpoint, facilitating its identification.

Additionally, as the total volume of titrant added increased, the overall ion concentration in the solution became more diluted, leading to a lower minimum conductivity. In theory, a smaller volume of titrant allows for a more complete and precise reaction, provided that the endpoint remains distinguishable. However, practical constraints such as the need for the solution to fully submerge the conductivity electrode impose a lower limit on the volume of the solution. Based on the experimental data, a sample volume of 5 mL was found to be optimal, offering clear endpoint identification while minimizing reagent consumption and maximizing test accuracy.

### 4.3. Effect of Titrant Concentration

The concentration of Na_2_SO_4_ directly affects the accuracy of SO_4_^2−^ titration. To investigate this effect, Na_2_SO_4_ solutions with concentrations of 0.01 mol/L, 0.005 mol/L, and 0.001 mol/L were titrated using Ba(NO_3_)_2_ solutions at 0.02 mol/L, 0.01 mol/L, and 0.005 mol/L, respectively. In all experiments, the volume of Na_2_SO_4_ was fixed at 5 mL. Details of the experimental setup are provided in Table 2.

**Figure 10:**
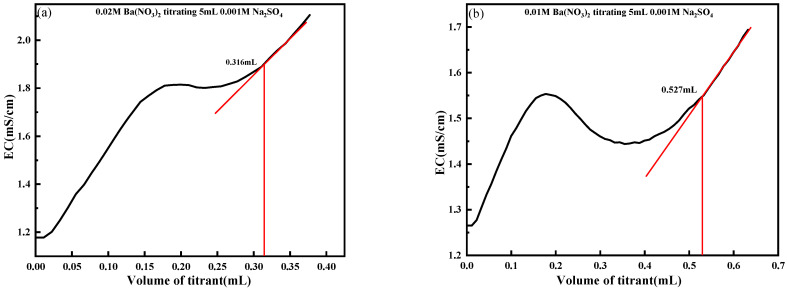

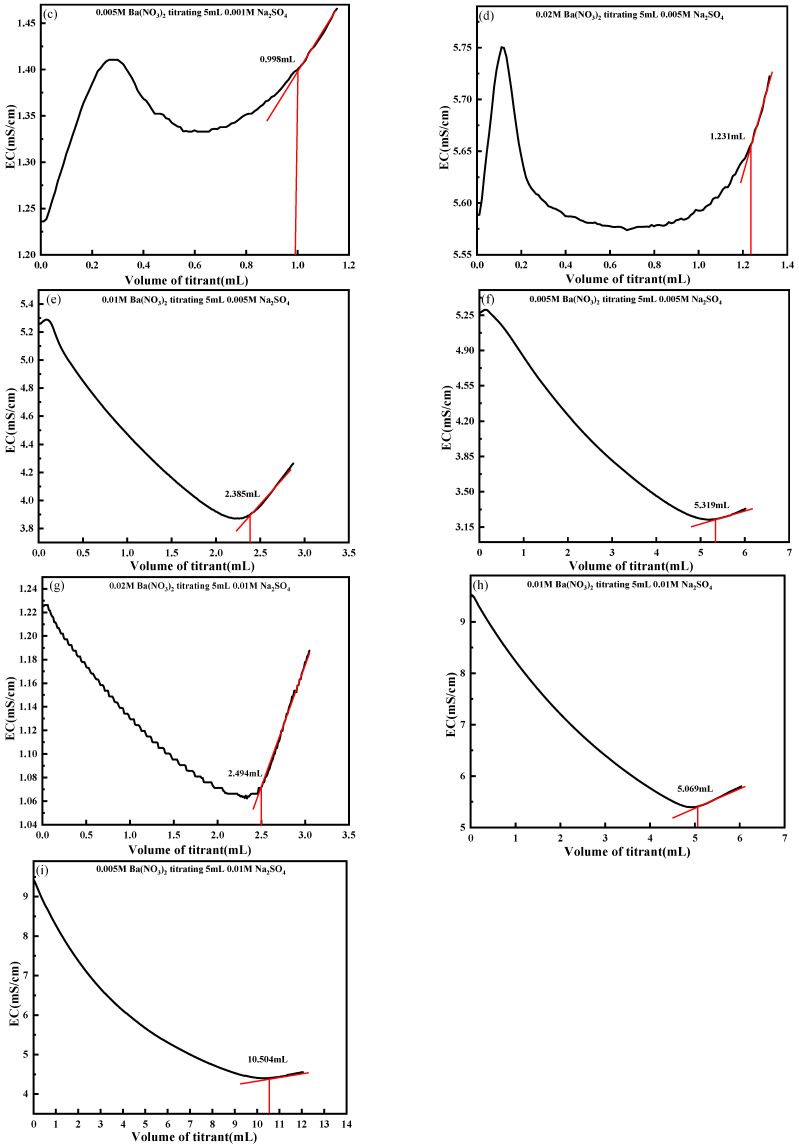
Influence of titrant concentration change on experimental accuracy. (**a**) 0.02 M Ba(NO_3_)_2_ titrating 0.001 M Na_2_SO_4_; (**b**) 0.01 M Ba(NO_3_)_2_ titrating 0.001 M Na_2_SO_4_; (**c**) 0.005M Ba(NO_3_)_2_ titrating 0.001M Na_2_SO_4_; (**d**) 0.02 M Ba(NO_3_)_2_ titrating 0.005 M Na_2_SO_4_; (**e**) 0.01M Ba(NO_3_)_2_ titrating 0.005 M Na_2_SO_4_; (**f**) 0.005 M Ba(NO_3_)_2_ titrating 0.005 M Na_2_SO_4_; (**g**) 0.02 M Ba(NO_3_)_2_ titrating 0.01 M Na_2_SO_4_; (**h**) 0.01 M Ba(NO_3_)_2_ titrating 0.01 M Na_2_SO_4_; (**i**) 0.005 M Ba(NO_3_)_2_ titrating 0.01 M Na_2_SO_4_.

As shown in Figure 10a, when 0.001 mol/L Na_2_SO_4_ was titrated with 0.02 mol/L Ba(NO_3_)_2_, the minimum addition of titrant introduced excess Ba^2+^ ions into the solution, causing the reaction to proceed too rapidly and making it difficult to determine the titration endpoint accurately. The experimentally measured endpoint volume was 0.316 mL, which significantly deviated from the theoretical value of 0.25 mL. Strong nonlinearity was observed in Figure 10a–d, particularly in Figure 10d, where the titrant concentration was high. This phenomenon was primarily attributed to two factors: delayed nucleation of BaSO_4_ due to precipitation kinetics, and an asymmetric conductivity response resulting from the replacement of Na^+^ by Ba^2+^, which differ in both charge and mobility. These effects distorted the expected linear relationship between conductivity and the titrant volume, especially during the early titration phase and near the endpoint. This nonlinearity underscored the necessity of optimizing titrant concentration to ensure reliable endpoint detection.

In Figure 10g, during the titration of 0.01 mol/L Na_2_SO_4_ with 0.02 mol/L Ba(NO_3_)_2_, the minimum titration volume contains a moderate quantity of barium ions. Thus, a notable change in conductivity occurs at the titration endpoint, facilitating the determination of the endpoint. The experimentally measured value is 2.494 mL, which approximates the theoretical value of 2.5 mL. As illustrated in Figure 10h, when 0.01 mol/L Ba(NO_3_)_2_ is used to titrate 0.01 mol/L Na_2_SO_4_, the conductivity curve exhibits relatively gentle fluctuations. The BaSO_4_ precipitate generated progressively during the titration interferes with the conductivity measurement, and the variation in conductivity at the endpoint is relatively slight. Determining the titration endpoint via the slope method proves challenging under these circumstances. In Figure 10i, when the concentration of Ba(NO_3_)_2_ is lower than that of Na_2_SO_4_, the titration process extends over an excessively long duration. The continuous precipitation of BaSO_4_ accumulates and may encapsulate reaction ions, creating unfavorable conditions for the determination of the titration endpoint.

In summary, to obtain more accurate titration results, Ba(NO_3_)_2_ with a concentration approximately twice that of the unknown Na_2_SO_4_ solution is recommended as the titrant. This selection ensures a significant change in conductivity at the titration endpoint while avoiding interference in measurement caused by excessive precipitate formation.

### 4.4. Effect of H^+^ on Conductivity

The determination of total sulfate concentration in PG and cement-based materials requires leaching sulfate from corrosion products using high concentrations of HCl or HNO_3_. The leaching solution contains a high level of H^+^, whose molar conductivity is significantly higher than that of other ions, thereby causing substantial interference in the conductivity titration process. However, accurate sulfate titration demands lower solution conductivity without reducing the sulfate concentration itself. Thus, investigating the impact of H^+^ on the conductivity of Na_2_SO_4_ solutions is essential.

Given the inherently low sulfate concentrations in sulfate-eroded concrete, this study utilizes 0.01 mol/L and 0.02 mol/L Na_2_SO_4_ solutions to evaluate the influence of H^+^ on solution conductivity. Two experimental groups were designed: Group A involved titrating 5 mL of 0.01 mol/L Na_2_SO_4_ with 0.02 mol/L Ba(NO_3_)_2_, and Group B involved titrating 5 mL of 0.02 mol/L Na_2_SO_4_ with 0.05 mol/L Ba(NO_3_)_2_. For both groups, two types of HCl additions were tested: the first type was 0.005 mol/L HCl, with an initial addition of 0.5 mL and subsequent increments of 0.5 mL, resulting in five additions (0.5 mL, 1.0 mL, 1.5 mL, 2.0 mL, and 2.5 mL) to reach a final volume of 2.5 mL; the second type was a single addition of 1 mL of 1 mol/L HCl. Each group was subjected to these six HCl addition protocols.

**Figure 11:**
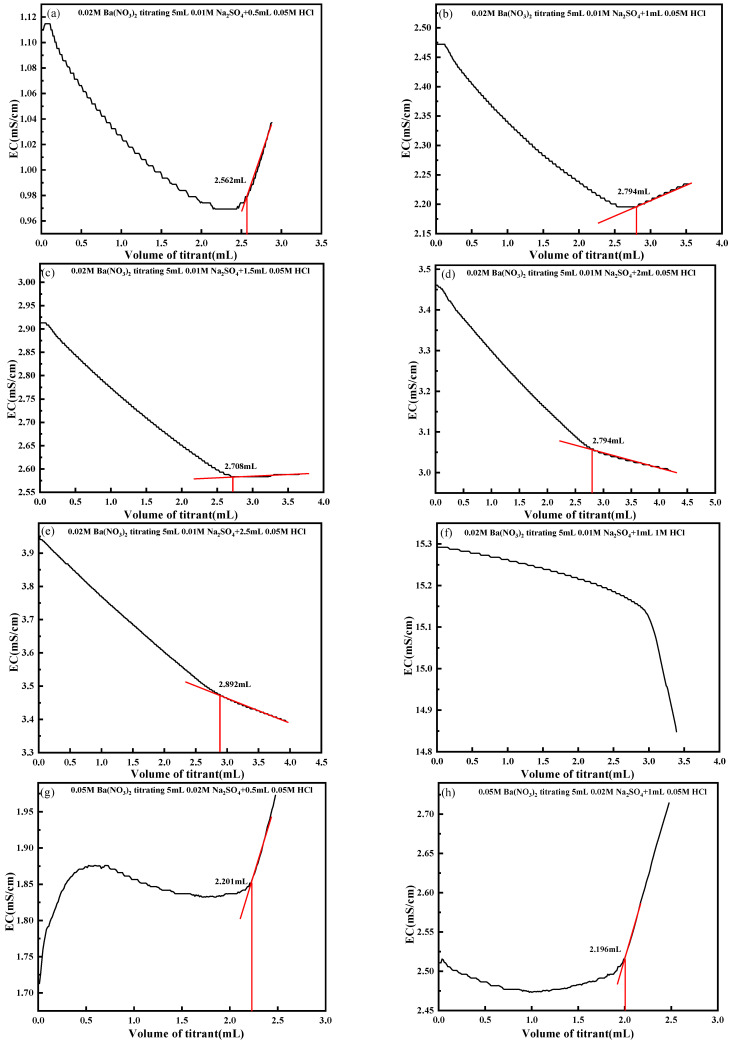

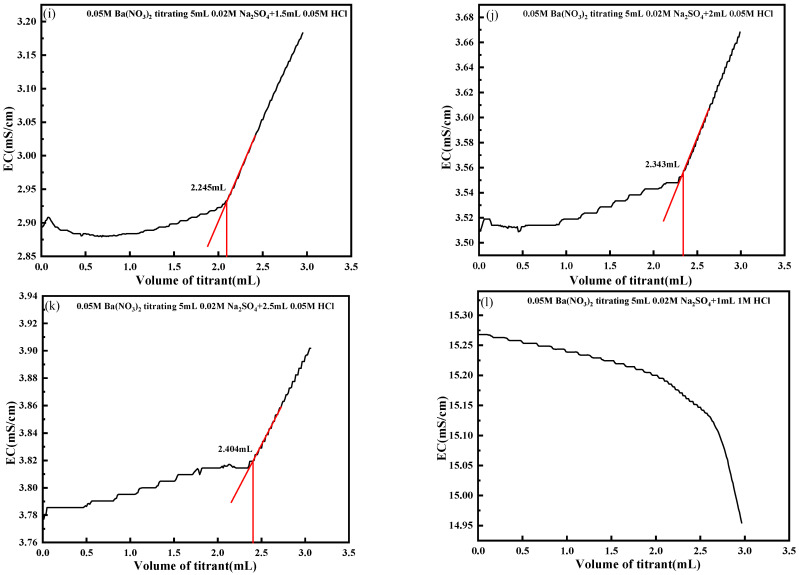
Effect of H^+^ on the conductivity titration of SO_4_^2−^. (**a**) With 0.5 mL 0.05 M HCl added; (**b**) With 1 mL 0.05 M HCl added; (**c**) With 1.5 mL 0.05 M HCl added; (**d**) With 2 mL 0.05 M HCl added; (**e**) With 2.5 mL 0.05 M HCl added; (**f**) With 1 mL 1 M HCl added; (**g**) With 0.5 mL 0.05 M HCl added; (**h**) With 1 mL 0.05 M HCl added; (**i**) With 1.5 mL 0.05 M HCl added; (**j**) With 2 mL 0.05 M HCl added; (**k**) With 2.5 mL 0.05 M HCl added; (**l**) With 1 mL 1 M HCl added.

Figure 11 demonstrates the effect of H^+^ on solution conductivity, and Table 3 presents the influence of H^+^ content in the solution on titration performance. For Group A, as illustrated in Figure 11a–f, H^+^ exerted a significant effect on the conductivity titration of SO_4_^2−^. When the volume of added HCl exceeded 1 mL, the accuracy of titration endpoint determination was impaired. Specifically, H^+^ reduced the proportion of SO_4_^2−^ contribution to solution conductivity, thereby weakening the conductivity decrease induced by the substitution of Cl^−^ for SO_4_^2−^ during titration. As shown in Figure 11f, the addition of 1 mL of 1 mol/L HCl caused severe disruption to the titration endpoint, making endpoint judgment impossible.

For Group B, as illustrated in Figure 11g–i, 0.02 mol/L Na_2_SO_4_ solutions were titrated with 0.05 mol/L Ba(NO_3_)_2_, with H^+^ concentrations adjusted via additions of HCl at varying volumes and concentrations. Experimental results confirm that H^+^ exerts a significant influence on the conductometric titration of SO_4_^2−^. When the volume of added HCl exceeds 1 mL, the titration endpoint determination becomes markedly erroneous. During titration, H^+^ interference complicates the conductivity trend of the solution, impeding accurate data acquisition. In environments with lower H^+^ concentrations, the impact of H^+^ on experimental data is approximately 10%. However, when H^+^ concentrations reach a critical threshold, such as upon addition of 1 mL of 1 mol/L HCl, the conductivity curve exhibits significant distortion. This severe distortion substantially impairs titration endpoint determination, rendering the endpoint indistinguishable.

### 4.5. Elimination of H^+^, Cl^−^ and Ca^2+^

In the chemical analysis of cement-based materials, accurately determining the SO_4_^2−^ content is essential for assessing material performance and durability. The influx of large amounts of SO_4_^2−^ ions into concrete induces a series of expansion reactions, with the following main reactions occurring:

(5) $${Ca\left(OH\right)}_{2}+{SO}_{4}^{2-}\to CaSO_{4}+2OH^{-}$$

(6) $$C_{3}A\cdot 3H_{2}O+3SO_{4}^{2-}+26H_{2}O\to {Ca}_{6}{Al}_{2}{\left({SO}_{4}\right)}_{3}\cdot 32H_{2}O$$

(7) $$C_{3}A\cdot 3H_{2}O+SO_{4}^{2-}\to C_{4}AS\cdot 12H_{2}O$$

The expansion products, such as CaSO_4_ and CaO, generated by the above reaction, will create stress concentrations in the micropore structure, eventually leading to the deterioration and cracking of concrete. To detect SO_4_^2−^ in concrete, traditional methods often utilize nitric acid or HCl to dissolve the powder; however, Cl^−^ is more easily removed by precipitation than NO_3_^−^. Therefore, we selected 1 mol/L HCl to extract SO_4_^2−^ in this study. A series of reactions accompany the extraction process:

(8) $${Ca\left(OH\right)}_{2}+2H^{+}\to {Ca}^{2+}+2H_{2}O$$

(9) $${CaCO}_{3}+2H^{+}\to {Ca}^{2+}+H_{2}O+{CO}_{2}↑$$

(10) $${CaSO}_{4}\to {Ca}^{2+}+{SO}_{4}^{2-}$$

(11) $$3{CaO\cdot Al}_{2}O_{3}{\cdot 2CaSO}_{4}\cdot {32H}_{2}O+H^{+}\to {Al\left(OH\right)}_{4}^{-}+{Ca}^{2+}+{SO}_{4}^{2-}+H_{2}O$$

(12) $$C-S-H+H^{+}\to {Ca}^{2+}+H_{3}{SiO}_{4}^{-}+H_{2}O$$

(13) $$C_{4}AF+H^{+}\to {Ca}^{2+}+{Fe}^{3+}+H_{3}{SiO}_{4}^{-}+H_{2}O$$

In the above reaction, some of the H^+^ in HCl reacts with CaCO_3_, C-S-H, and Ca(OH)_2_ to form Ca^2+^, but excess H^+^ remains in the solution after this reaction. Effective removal of interfering ions from the solution is necessary to enhance the contribution of SO_4_^2−^ to the solution’s conductivity and better assess the titration endpoint.

The combination of Ag_2_O and continuous CO_2_ passage can effectively decrease the concentrations of H^+^, Cl^−^, and Ca^2+^ in the solution, thereby reducing the interference of these ions on the SO_4_^2−^ contribution to conductivity. Firstly, upon adding Ag_2_O to the solution, some H^+^ reacts with Ag_2_O to generate water molecules, decreasing conductivity. This reaction causes the conductivity to trend downward. Simultaneously, the Cl^−^ in the solution reacts with Ag^+^ released from dissolving Ag_2_O to produce water-insoluble AgCl precipitate, effectively removing Cl^−^. With the addition of Ag_2_O, the following reactions occur:

(14) $${Ag}_{2}O+2H^{+}⇋2{Ag}^{+}+H_{2}O$$

(15) $${Ag}^{+}+{Cl}^{-}⇋AgCl↓$$

When the H^+^ in the solution is completely consumed, the solution gradually changes from acidic to neutral. At this point, Ag_2_O undergoes a hydrolysis reaction in aqueous solution. The Ag^+^ produced by hydrolysis continues to combine with Cl^−^ in solution to produce an AgCl precipitate.

(16) $${Ag}_{2}O+H_{2}O⇋{Ag}^{+}+2{OH}^{-}$$

(17) $${Ag}^{+}+{Cl}^{-}⇋AgCl↓$$

The hydrolysis of Ag_2_O changes the nature of the solution from neutral to basic. Since a large amount of Ca^2+^ is still present in the solution, a large amount of CaCO_3_ precipitate is produced when CO_2_ is passed into the solution under test.

(18) $$2{OH}^{-}+{Ca}^{2+}+{CO}_{2}⇋{CaCO}_{3}↓+H_{2}O$$

Upon injection of CO_2_, the solution will change from basic to neutral. Ag^+^ and OH^−^ can be generated again by adding additional Ag_2_O or by shaking the Ag_2_O-containing solution so that it continues to hydrolyze. In this case, Ag^+^ and Cl^−^ will continue to react to produce an AgCl precipitate. After the above step-by-step treatment, the concentrations of H^+^, Cl^−^, and Ca^2+^ in the solution under test are significantly reduced. The supernatant can then be taken for neutralization and conductivity titration of SO_4_^2−^.

As a sparingly soluble solid, Ag_2_O tends to have AgCl precipitates adhering to its surface, which inhibits further hydrolysis. Ultrasonic cleaning is therefore recommended to remove surface-bound AgCl, enabling sustained reaction or hydrolysis of Ag_2_O.

To investigate the effect of the content of H^+^, Cl^−^, and Ca^2+^ in the solution on the conductivity of the solution and to verify the effectiveness of the synergistic method in removing the interfering ions, three sets of experiments were set up by preparing 5 mL of Na_2_SO_4_ at a concentration of 0.01 mol/L as the baseline solution and preparing HCl at a concentration of 0.1 mol/L and CaCl_2_ at a concentration of 0.015 mol/L as the experimental solution. A CO_2_ generator generated CO_2_ and was continuously fed during the reaction.

**Figure 12:**
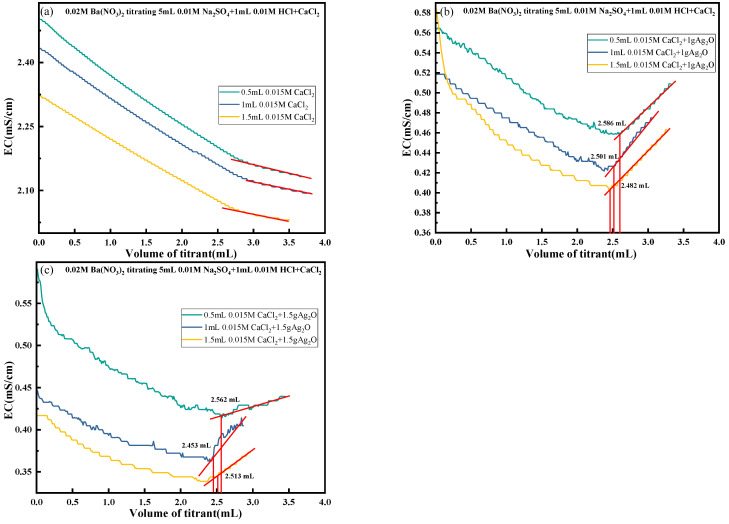
Results of titration under elimination of H^+^, Cl^−^, and Ca^2+^ from solution to be tested. (**a**) No Ag_2_O added; (**b**) With 1g Ag_2_O added; (**c**) With 1.5g Ag_2_O added.

The concentration of Na_2_SO_4_ was controlled to remain constant during the experiment, and a specific volume and concentration of CaCl_2_ and HCl were added, and the amount of Ag_2_O varied. The results of the experiment are shown in Figure 12. As can be seen from Figure 12b, when the amount of Ag_2_O was 1 g, the titration endpoint of the solution was easy to judge, and the actual values obtained from the titration were 2.586, 2.501, and 2.482 mL, respectively, close to the theoretical value of 2.5 mL. It demonstrates that an appropriate amount of Ag_2_O can effectively remove Cl^−^ and H^+^ from the solution, reducing their interference with solution conductivity. This enhancement in SO_4_^2−^ contribution to solution conductivity improves the accuracy of titration endpoint determination. Figure 12c shows that when the Ag_2_O amount was 1.5 g, the actual titration values were 2.562, 2.453, and 2.513 mL, respectively, still close to the theoretical 2.5 mL. However, excess Ag_2_O addition caused the curve change to become non-smooth, complicating endpoint determination. This phenomenon may arise because excess Ag_2_O, after reacting with H^+^ to form Ag⁺ ions, further reacts with water to hydrolyze, generating additional Ag⁺ and OH^−^. The increased OH^−^ concentration raises the solution pH, and under weakly alkaline conditions, SO_4_^2−^ reacts with Ag^+^ to form soluble complexes, thereby altering solution conductivity.

The precipitates obtained from the titration of the composite solution after pretreatment were analyzed using XRD and SEM to verify the rationality of the synergistic method for removing H^+^, Ca^2+^, and Cl^−^ from the solution. SEM tests were performed using a 3D FEG SEM from the FEI company, equipped with an EDAX Genesis energy spectrometer, and with NanoSEM450 from the AMETEK company. The accelerating voltage of the electron gun was 15 kV. The target material was platinum.

**Figure 13:**
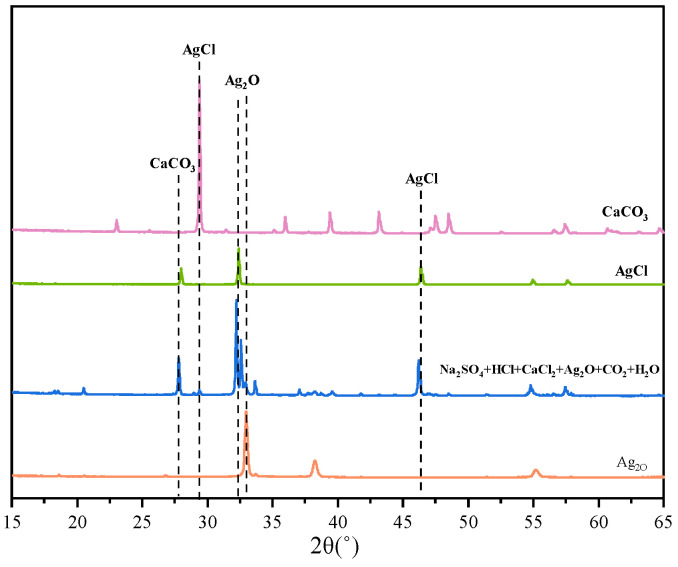
XRD of precipitation after treatment to eliminate H^+^, Ca^2+^, and Cl^−^.

**Figure 14:**
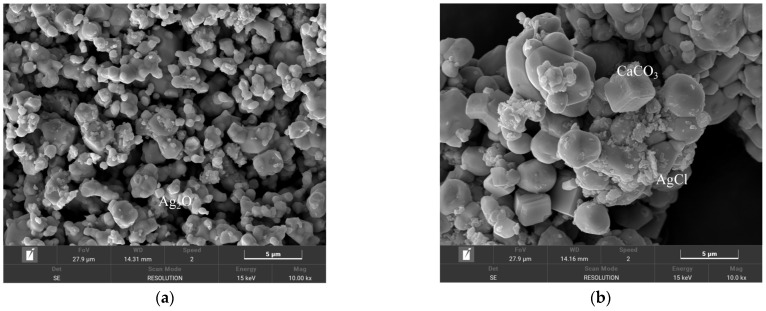
SEMs of precipitation after pretreatment to eliminate H^+^ and Cl^−^ by adding Ag_2_O and CO_2_. (**a**) SEM Image of Ag_2_O Precipitate; (**b**) SEM Image of CaCO_3_ and AgCl Precipitates.

As can be seen from Figure 13, the precipitates obtained by titration after treatment of solutions containing H^+^, Ca^2+^, and Cl^−^ are by the theory, and only AgCl and CaCO_3_ precipitates were produced in the test solutions containing H^+^, Ca^2+^, and Cl^−^. The precipitate SEM after pretreatment by adding Ag_2_O and CO_2_ to remove H^+^, Ca^2+^, and Cl^−^ is shown in Figure 14. Fine AgCl particles were present in the precipitates, and Ag_2_O in the precipitates was difficult to observe due to the covering of AgCl. In addition, CaCO_3_ in the cubic style was present between the precipitates, and no other precipitates were generated, except for AgCl and CaCO_3_.

### 4.6. Determination of SO_4_^2−^ Concentration in PG and Cement-Based Materials by Conductivity Titration

Directly detecting SO_4_^2−^ concentration in cement-based materials using embedded sensors is not feasible. Instead, sulfate must be extracted from the hardened cementitious materials before it can be detected. The process of determining SO_4_^2−^ content in cementitious materials via conductivity titration involves several key steps: powder extraction, dissolution, filtration, and titration.

Experimentally prepared mortar specimen blocks with dimensions of 100 mm × 100 mm × 100 mm, a water–cement ratio of 0.5, a sand–cement ratio of 3, a 7 d compressive strength of 32.47 MPa, and a 28 d compressive strength of 37.66 MPa were tested by immersing the prepared mortar specimen blocks in a 5% Na_2_SO_4_ solution for 9 months.

**Figure 15:**
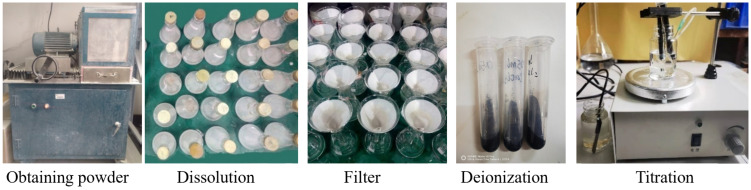
Titration procedure.

The cement-based materials subjected to sulfate leaching were graded and ground using a powder extractor at grinding depths of 0–2 mm, 2–4 mm, 4–6 mm, 6–8 mm, 8–10 mm, and 10–12 mm, The ground powder with a particle size of less than 0.075 mm was obtained through sieving. For each depth, 3 g of the powder was immersed in 30 mL of 1 mol/L HCl for 24 h. A vibrating sieve was used to accelerate the dissolution of SO_4_^2−^ in the material. The leachate was deionized through slow filter paper, and 5 mL of the filtrate was titrated to detect sulfate. The Ba(NO_3_)_2_ concentration used in the titration was 0.05 mol/L, and 1.5 g of Ag_2_O was added. The titration procedure is shown in Figure 15.

**Figure 16:**
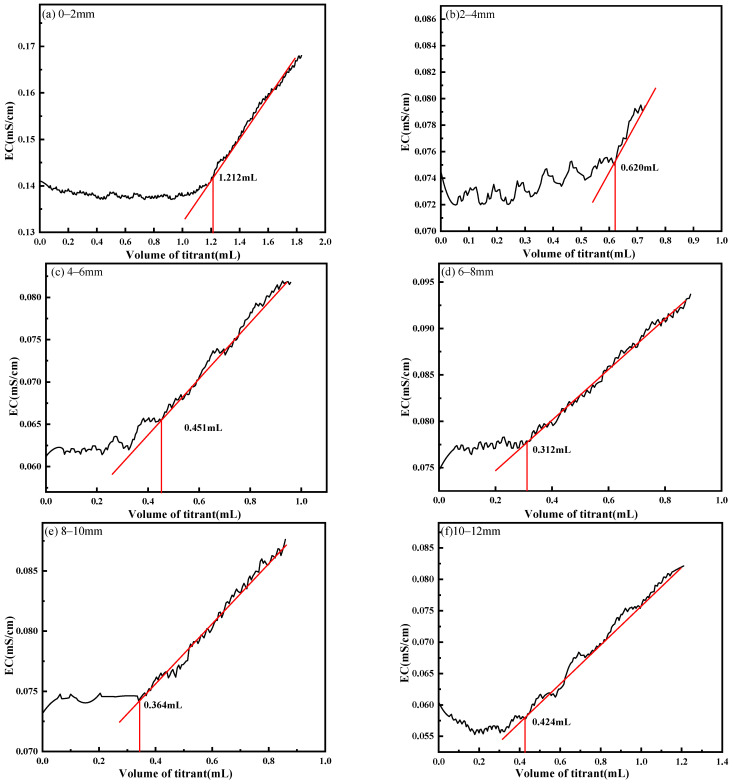
Determination of sulfate content in cement-based materials at different depths by conductometric titration. (**a**) 0–2 mm; (**b**) 2–4 mm; (**c**) 4–6 mm; (**d**) 6–8 mm; (**e**) 8–10 mm; (**f**) 10–12 mm.

The conductivity titration curves of SO_4_^2−^ in a 0–12 mm thickness mortar subjected to a sulfate attack and its titration endpoint are shown in Figure 16. The sulfate content in the cementitious materials at different grinding depths is different; the conductivity of the solution decreases with the increase in the depth of corrosion, and the volume of barium nitrate consumed in the titration with the increase in the grinding solubility is 1.212, 0.620, 0.451, 0.312, 0.364, and 0.424 mL, respectively. The sulfate content of the samples measured was 1.16%, 0.60%, 0.43%, 0.30%, 0.35%, and 0.41%. The sulfate content of the samples decreases with the increasing grinding depth, with a 64% decrease from 10 to 12 mm depth of grinding compared to 0 to 2 mm depth of grinding.

**Figure 17:**
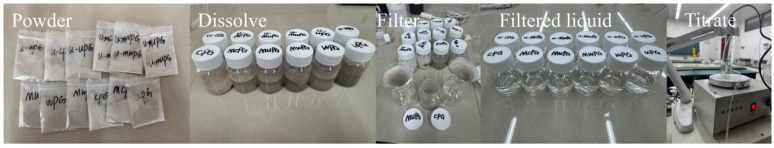
Main steps of titration of PG-based cementitious materials.

Many harmful impurities, including soluble phosphorus, eutectic phosphorus, soluble fluorine, and organic matter, complicate the direct utilization of PG. However, PG holds significant potential for development as an engineering material. It has been used as a raw material in various applications, including high-performance concrete [29], gypsum board manufacturing [30], retardant production, and lime manufacturing [31]. To evaluate the practicality of the self-developed conductivity titrator and assess the feasibility of reusing PG, the sulfate ion content in six different PG-based gelling materials was determined. The main steps involved in the determination included five stages: powder extraction, dissolution, filtration, and titration, as illustrated in Figure 17.

Firstly, the PG-based cementitious material was graded and ground using a powder extractor, and the ground powder with a particle size of less than 0.075 mm was obtained through a sieve; m1 g of the powder was immersed in V_1_ mL of dilute HCl for 24 h, and a vibrating sieve was used at the same time to accelerate the solubilization of SO_4_^2−^ in the material; the leaching solution was filtered through a slow filter paper, and V_2_ mL of the filtrate was taken for titration for the detection of sulfate. The concentration of Ba(NO_3_)_2_ used for the titration was ${C_{Ba{(NO}_{3})}}_{2}$. When the titration reached the endpoint, the volume of Ba(NO_3_)_2_ consumed was recorded as X mL.

**Figure 18:**
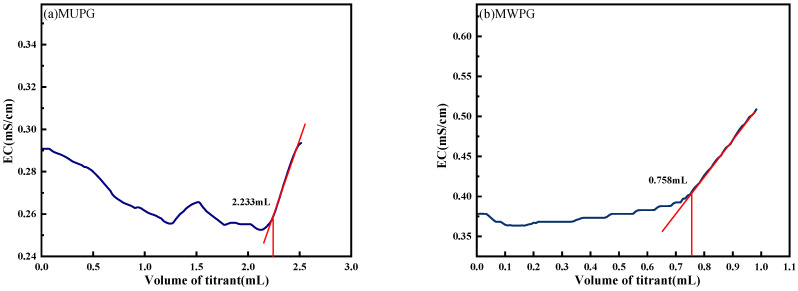

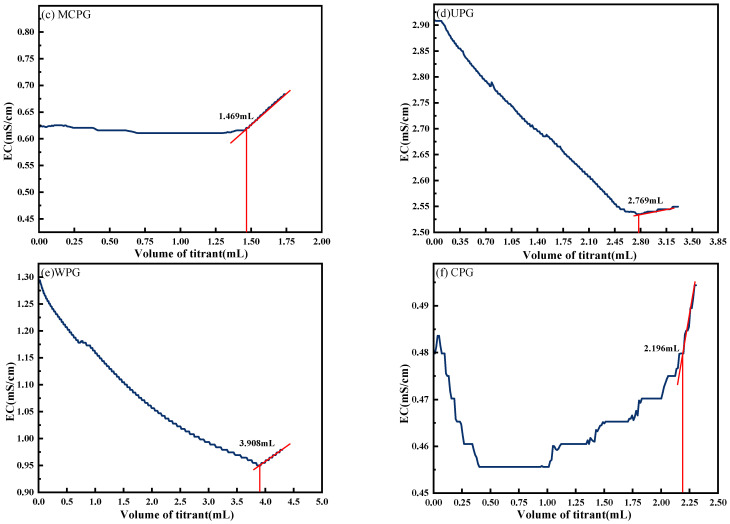
Titration of different types of PG-based cementitious materials. (**a**) MUPG; (**b**) MWPG; (**c**) MCPG; (**d**) UPG; (**e**) WPG; (**f**) CPG.

In this experimental study, the grinding thickness of PG-based cementitious material was 2 mm, the mass of powder used for leaching was 2 g, the weighing accuracy was 0.0001 g, the volume was 20 mL, the volume of titration solution was 5 mL, and the concentration of Ba(NO_3_)_2_ was 0.05 mol/L. The titration results are shown in Figure 18, where the volume of Ba(NO_3_)_2_ solution consumed for titration, measured by conductometric titration, was different for different samples in this experiment. For the test samples, M stands for hydrophobically modified, UPG for as-received phosphogypsum, WPG for washed phosphogypsum, and CPG for calcined phosphogypsum. The volumes of titrant consumed for each sample, according to Figure 18a–f, were 2.233, 0.758, 1.469, 2.769, 3.908, and 2.196 mL.

## 5. Conclusions

This study proposed a self-developed high-precision conductometric titration system, systematically evaluated, and ultimately used for the quantitative determination of SO_4_^2−^ in cement-based and PG-based materials. This system facilitates the sustainable utilization of industrial by-products such as phosphogypsum and provides a promising analytical tool for assessing the durability of concrete in sulfate-containing environments. Through systematic research, the following conclusions can be obtained:

1. A conductivity titration system based on the Goouuu-ESP32 microcontroller was developed for the quantitative detection of sulfate ions. Experimental validation yielded an average Ba(NO_3_)_2_ titration volume of 2.498 mL, with a standard deviation of 0.013 mL, a coefficient of variation of 0.52%, an average relative error of 0.32%, and recovery rates ranging from 103.2% to 103.9%, indicating excellent accuracy and reproducibility of the system.
2. The influence of key titration parameters, including the solution volume and titrant concentration, was comprehensively analyzed. The results suggest that a test solution volume of 5 mL and a Ba(NO_3_)_2_ concentration approximately twice that of the SO_4_^2−^ content yielded optimal endpoint clarity. These optimized conditions enhance conductivity responses and mitigate endpoint ambiguity caused by excess or insufficient titrant addition.
3. To address the interference of high-conductivity ions such as H^+^, Cl^−^, and Ca^2+^, a synergistic elimination method involving Ag_2_O and CO_2_ was developed. This approach effectively reduced ionic interference by promoting the formation of weakly conductive precipitates, including AgCl and CaCO_3_, thereby restoring the dominant contribution of SO_4_^2−^ to solution conductivity and ensuring accurate endpoint detection.
4. The leaching–filtering–treatment of the filtration–titration process accurately detected SO_4_^2−^ in the sulfate determination of cement-based and PG-based materials, which verified the applicability of the method for industrial solid waste resource utilization and concrete durability assessment.

## Figures and Tables

**Table 1:** Experimental data for recycling rate.

Group	Initial Concentration (mol/L)	Spiked Concentration (mol/L)	Theoretical Volume Increment (mL)	Actual Volume Increment (mL)	Recovery Rate (%)
A	0.1	0.02	2	2.079	103.9
B	0.2	0.01	1	1.032	103.2

**Table 2:** Experimental setup for titrant concentration change.

Group	Titrant Concentration (mol/L)	Concentration of the Test Solution (mol/L)
A	0.02/0.01/0.005	0.001
B	0.02/0.01/0.005	0.005
C	0.02/0.01/0.005	0.010

**Table 3:** Effect results of H^+^ on the conductivity titration of SO_4_^2−^.

Group	HCl Concentration (mol/L)	Volume of HCl (mL)	Theoretical Titration Volume (mL)	Actual Titration Volume (mL)	Impact Rate (%)
A	0.05	0.5	2.500	2.562	2.48
	0.05	1.0	2.500	2.794	11.76
	0.05	1.5	2.500	2.708	8.32
	0.05	2.0	2.500	2.794	11.76
	0.05	2.5	2.500	2.892	15.68
	1.00	1.0	2.500	-	-
B	0.05	0.5	2.000	2.201	10.05
	0.05	1.0	2.000	2.196	9.80
	0.05	1.5	2.000	2.245	12.25
	0.05	2.0	2.000	2.343	17.15
	0.05	2.5	2.000	2.404	20.20
	1.00	1.0	2.000	-	-

## Data Availability

The original contributions presented in this study are included in the article. Further inquiries can be directed to the corresponding author.
